# Predominance of Norovirus and Sapovirus in Nicaragua after Implementation of Universal Rotavirus Vaccination

**DOI:** 10.1371/journal.pone.0098201

**Published:** 2014-05-21

**Authors:** Filemón Bucardo, Yaoska Reyes, Lennart Svensson, Johan Nordgren

**Affiliations:** 1 Department of Microbiology, University of León (UNAN-León), León, Nicaragua; 2 Division of Molecular Virology, Department of Clinical and Experimental Medicine, Linköping University, Linköping, Sweden; National Taiwan University Hospital, Taiwan

## Abstract

**Background:**

Despite significant reduction of rotavirus (RV) infections following implementation of RotaTeq vaccination in Nicaragua, a large burden of patients with diarrhea persists.

**Methods:**

We conducted a community- and hospital-based study of the burden of RV, norovirus (NV) and sapovirus (SV) infections as cause of sporadic acute gastroenteritis (GE) among 330 children ≤ 5 years of age between September 2009 and October 2010 in two major cities of Nicaragua with a RotaTeq coverage rate of 95%.

**Results:**

We found that NV, SV and RV infections altogether accounted for 45% of cases of GE. Notably, NV was found in 24% (79/330) of the children, followed by SV (17%, 57/330) and RV (8%, 25/330). The detection rate in the hospital setting was 27%, 15% and 14% for NV, SV and RV respectively, whereas in the community setting the detection rate of RV was < 1%. Among each of the investigated viruses one particular genogroup or genotype was dominant; GII.4 (82%) for NV, GI (46%) for SV and G1P[8] (64%) in RV. These variants were also found in higher proportions in the hospital setting compared to the community setting. The GII.4.2006 Minerva strain circulating globally since 2006 was the most common among genotyped NV in this study, with the GII.4-2010 New Orleans emerging in 2010.

**Conclusions:**

This study shows that NV has become the leading viral cause of gastroenteritis at hospital and community settings in Nicaragua after implementation of RV vaccination.

## Introduction

Acute gastroenteritis (GE) is one of the leading causes of morbidity and mortality in children in the developing countries, with rotavirus (RV) and norovirus (NV) being the major causes of pediatric viral GE, altogether associated with approximately 800,000 deaths in young children every year [1]–[3]. In contrast, sapovirus (SV) infections are more rarely reported and generally considered to cause milder symptoms, with detection rates rarely reaching 10% [4]–[8].

A major reduction of severe RVGE has been observed in countries with high vaccine coverage but comprehensive analysis in several clinical trials indicates the vaccine efficacy to be lower in countries with high RV mortality [9], [10]. In October 2006, the Nicaraguan Expanded Program of Immunization (EPI) initiated universal RV vaccination with the pentavalent RV vaccine from Merck (RotaTeq), which is orally administrated in a 3-dose regimen to children at 2, 4, and 6 months of age. Vaccine coverage rapidly reached over > 90% in eligible Nicaraguan children [11]. A case control study evaluating RotaTeq in 2007–2008 in Nicaragua showed that vaccination was associated with a lower risk of RVGE in children younger than 2 years [12], but to a lesser extent than observed in clinical trials in Europe and USA [13]. The efficacy in Nicaragua against severe RV diarrhea was only 58%, which is similar to other studies from Asian and African countries [12], [14], [15]. In the post-vaccine period, a study found that 3.5% of outpatient children seeking care for diarrhea were positive for RV [16]. However, while RV incidence has decreased in Nicaragua, the overall number of GE cases of any etiology has remained at high levels after RV vaccine introduction [17]. A recent paper reports that a pre-vaccine community cohort experienced 36 episodes of watery diarrhea per 100 child-years, whereas a vaccine cohort experienced 25 episodes per 100 child-years, indicating a 60% persistence of watery diarrhea at community level during the vaccine era [18].

A non-specific effect of RV vaccine on GE of other etiologies has been suggested in a few studies [19]–[21]. However, this effect is still speculative and whether RV vaccination has any effect on NVGE or SVGE remains to be established.

In this study we investigated the virological causes behind the burden of diarrhea that persists after implementation of universal RV vaccination in Nicaragua. We thus explored the relative frequency of NV, SV and RV, both at hospital and community level in Nicaragua during 2009/2010 in the context of high RV vaccination rates. In addition we explored the association of these relative frequencies with RV vaccination status, dehydration status, and virus genotypes/genogroups.

## Materials and Methods

### Ethics statement

The study protocol and consent procedure was approved by the local ethical committee for biometrics research (Comite de Etica para lnvestigaciones Biomedicas [CEIB]; registration number: 61-2005, 73-2010). Informed written consent was given by parents or child guardians before samples were collected as described below. After the consent was given, personal details, as well as, epidemiological data were recorded in a paper file.

### Study Design

Through a community- and hospital-based passive surveillance of sporadic acute diarrhea, a total of 330 children of ≤ 5 years of age with acute diarrhea were enrolled in a longitudinal, prospective manner from September 2009 to October 2010. In the hospital setting children (n  =  175) were enrolled at the pediatric wards and emergency rooms from main hospitals of León and Jinotega, Nicaragua (Hospital Escuela “Oscar Danilo Rosales and Victoria Motta, respectively). Community children (n  =  155) were outpatients consulting for diarrhea at two clinics of León.

### Clinical assessment

All children involved in the study were clinically evaluated by pediatricians (hospital) or general practitioners (community) and classified by dehydration status, following the WHO protocol for integrated management of childhood illness (IMCI), into one of the following categories: “severe-dehydration”, “some-dehydration”, and “without-dehydration”.

### RotaTeq immunization assessment

The dates each child received vaccine doses in Nicaragua were registered by an EPI nurse on the child's vaccination-card. The RotaTeq immunization data used in this study were collected from the children's vaccination-cards. A child was considered “unvaccinated” if their vaccination card showed no recorded doses. If the child's vaccination card was not available, immunization status was considered unknown.

### Collection of stool specimens

Fecal specimens were collected in sterile containers ≤ 24 h after admission, and transported either weekly (Jinotega) or daily (León) at 4°C to the microbiology laboratory of UNAN-León. At the two outpatient clinics, fecal samples were collected by a nurse during the visit. Before transportation, samples were stored at 4°C at the collection site to ensure quality of virological testing. A 10% (wt/vol) suspension of stool material was prepared with phosphate-buffered saline (pH  =  7.2), and two aliquots were frozen at −20°C for later examination of RV, NV and SV.

### RV antigen detection

A direct enzyme immunoassay for detection of RV in fecal specimens, OXOID ProSpecT R240396 (Cambridge, UK), was used according to the manufacturer's instructions. The results were visually read and confirmed by absorbance measurements.

### RNA extraction from stool specimens

Viral RNA was extracted from stool suspensions (200 µl) following the manufacturer's instructions using High Pure Viral RNA Kit (Roche Applied Science, Indianapolis, USA). A total of 50 µl of RNA was collected and stored at −20°C until reverse transcription (RT).

### Reverse transcription

Reverse transcription (RT) was carried out as described previously [22]. Briefly, 28 µl of purified RNA was mixed with 50 pmol of random hexadeoxynucleotides [pd(N)6] (GE Healthcare Life Sciences), and the mixture was denatured at 97°C for 5 min and quickly chilled on ice for 2 min, followed by the addition of one RT bead (Amersham Biosciences, UK) and RNase-free water to a final volume of 50 µl. RT reaction mixtures were incubated for 30 min at 42°C to produce complementary DNA (cDNA).

### RV G and P multiplex genotyping

The G and P genotypes of RVs recovered from stool samples were determined by PCR. The generic and genotype-specific primers used for detecting VP7 genotypes G1, G2, G3, G4, G8, G9, G10, and G12 were described previously [23], [24]. Primers used for detecting VP4 genotypes P[4], P[6], P[8], and P[9] were also described previously [23], [25]. Genotypes and full genomic analysis of RV found in RotaTeq vaccinated children from Jinotega has been described previously [26].

### NV detection by real-time PCR

In brief, 2.5 µl of purified RNA was added to a reaction mixture consisting of 12.5 µl of FastStart Universal SYBR Green Master (ROX) (Roche Applied Science, IN, USA), 1 µl (10 pmol) of each non-labeled GI and GII primers (NVG1f1b and NVG1rlux, NVG2flux1 and COG2R), [27] and 8 µl of RNAse free water, to final volume of 25 µl. The real-time PCR reactions were performed in a 96-well reaction plate using the ABI 7500 Real Time PCR System (Applied Biosystems, Foster, CA). PCR was performed under the following conditions: 95°C for 5 min, followed by 40 cycles of 95°C for 15 s, 55°C for 30 s and 72°C for 1 min. Melting curve analysis, to confirm amplicon specificity, was performed immediately after PCR completion.

### NV GI and GII genogrouping

All NV-positive samples were re-analyzed by genogroup specific SYBR Green real-time PCR, in separate tubes, following the procedure described for NV screening but using specific primers for either GI or GII [27]. A sample was considered of NV GI and/or GII if the Ct value was ≤ 40 and Tm of 76.1 ± 0.6°C for GI and 77.1 ± 0.6°C for GII.

### NV genotyping

A subset of NV-positive samples with sufficient viral load were sequenced in the N-terminal region of the capsid gene as described [22]. The 380-bp and 378-bp amplicons obtained from GI and GII NVs, respectively were sequenced by Macrogen Inc. (Seoul, South Korea)., using NVG1f1b, G1SKR, NVG2flux1 and G2SKR primers as sequencing primers [27], [28]. Sequence alignment was performed by using the ClustalW algorithm, version 1.83, with default parameters on the European Bioinformatics Institute server (EMBL-EBI). Phylogenetic analysis was performed using the MEGA 5.03 software package and the tree was constructed using the neighbor-joining method with the Kimura two-parameter model [29]. The significance of phylogenetic relationships was assessed by bootstrap resampling analysis (1,000 replications). The assignment of genotypes was done using pairwise nucleotide distance measurements [30].

### SV detection by real-time PCR

SV screening was carried out following a modification of the method described by Chan et al 2006 [31]. In brief, 2.5 µl of purified RNA was added to a reaction mixture consisting of 12.5 µl of 2X RT-PCR Buffer and 1 µl 25X RT-PCR Enzyme Mix (AgPath-ID One-Step RT-PCR kit, Life Technologies, NY, USA), 1 µl (10 pmol) of each forward (CUSVF1 and CUSVF2) and reverse primer (CUSVR), 1 µl (10 pmol) of SV TaqMan Probe (TaqMan MGB was replaced with [6FAM] TGG TTY ATA GGY GGT AC [BHQ1]), 1.67 µl of detection enhancer and 4.33 µl of RNAse free water, to final volume of 25 µl. The real-time PCR reactions were performed on a 7500 Real-Time PCR System (Applied Biosystems, Foster City, CA). A sample with Ct value ≤ 40 was considered positive.

### SV genogrouping

The cDNA of SV-positive specimens were amplified by an outer PCR using a primer pool that consisted of two forward (SV-F13 and SV-F14), and two reverse (SV-R13 and SV-R14) primers with PCR conditions as described elsewhere [32]. Genogroups, GI, GII, GIV and GV, were examined by nested PCR under identical conditions to the outer PCR using a primer pool that consisted of universal forward primers (SV-F13/-F14) and genogroup specific reverse primers (SV-G1-R, SV-G2-R, SV-G4-R and SV-G5-R). The 500-bp, 430-bp, 360-bp and 290-bp amplicons obtained from GI, GII, GIV and GV viruses, respectively, were visualized by 2% agarose gel electrophoresis followed by ethidium bromide staining. Co-infections with SV of different genogroups was confirmed by allele specific PCR.

### SV genotyping

The N-terminal region of the capsid gene (∼ 420 bp) was sequenced in a subset of 23 SV-positive samples. A total of 1 µl of PCR product from the outer PCR reaction, described in SV genogrouping section, 10 pmol of each of forward (F22) and reverse (R2) primers [32] and RNase-free water were added to one Illustra PuReTaq Ready-To-Go PCR Bead (GE Healthcare, Uppsala, Sweden) to a final volume of 25 µl. The PCR reaction was performed following the conditions described previously [32]. The 420-bp amplicons obtained from SV-positive samples were sequenced by Macrogen Inc. (Seoul, South Korea) using F22 and R2 primers as sequencing primers. The assignment of genotypes was done using pairwise nucleotide distance measurements.

### Statistical analysis

Relative frequencies between viral etiologies and genetic variants were analyzed for setting, clinical and epidemiological variables to investigate trends or statistical differences. Unadjusted Odds ratios (OR) were used to determine the degree of association and Fisher's exact test was used to compare frequencies of qualitative variables. Differences were considered to be statistically significant when the level of two-tailed significance was < 0.05.

The softwares SPSS (Statistical Program for Social Science version 14.0 for Windows; Chicago, IL) and GraphPad Prism (version 5.00 for Windows, GraphPad Software, San Diego California USA) were used for statistical analysis.

### Nucleotide sequence accession numbers

The nucleotide sequences for NV, SV and RV determined in this work were submitted to GenBank and the following accession numbers were assigned for Norovirus: KF361389 to KF361442; Sapovirus: KF361366 to KF361388 and Rotavirus: JN129124, JN129096, JN129110, JN129054, JN129068, JN129082, JN128984, JN128998, JN129012, JN129026, JN129040.

## Results

### High detection rates of viruses among children with diarrhea vaccinated with RotaTeq

In total, viral GE was associated with 147 (45%) of the 330 diarrheal children investigated. The RV vaccine coverage observed in the current study was 95% (270/284; Table 1). Of these, 213 (79%), 43 (16%) and 14 (5%) had received 3, 2 and 1 vaccine dose(s) respectively. For 46 children, the vaccination status was not known.

**Table 1:** Epidemiological profile and dehydration status of norovirus, sapovirus and rotavirus infections in a population with high RotaTeq coverage in Nicaragua, 2009–2010.

Parameter	Total	Norovirus n (%)	Sapovirus n (%)	Rotavirus n (%)
**All children**	330^a^	79 (24)	57 (17)	25 (8)
**Site**				
León	227	57 (25)	44 (19)	8 (4)
Jinotega	103	22 (21)	13 (13)	17 (16)
**Setting**				
Hospital	175	47 (27)	27 (15)	24 (14)
Community	155	32 (21)	30 (19)	1 (1)
**Gender**				
Male	190	45 (24)	34 (18)	13 (7)
Female	140	34 (24)	23 (17)	12 (9)
**Age range (mo)**				
≤ 6	64	16 (25)	8 (12)	4 (6)
7–12	108	31 (29)	21 (19)	9 (8)
13–24	107	25 (23)	24 (22)	7 (7)
25–60	51	7 (14)	4 (8)	5 (10)
**RV vaccination status**				
Vaccinated	270	64 (24)	51 (19)	16 (6)
Unvaccinated	14	3 (21)	0 (0)	2 (14)
Unknown	46	12 (26)	6 (13)	7 (15)
**Dehydration status**				
Severe dehydration^b^	57	15 (26)	9 (16)	16 (28)
Some dehydration	98	28 (29)	14 (14)	7 (7)
No dehydration	175	36 (21)	34 (19)	2 (1)

a Detection rates of norovirus, sapovirus and rotavirus is calculated per line in the table; thus for the total number of children per parameter

b All children with severe dehydration required intravenous rehydration.

### Norovirus was the major cause of pediatric gastroenteritis

**Figure 1:**
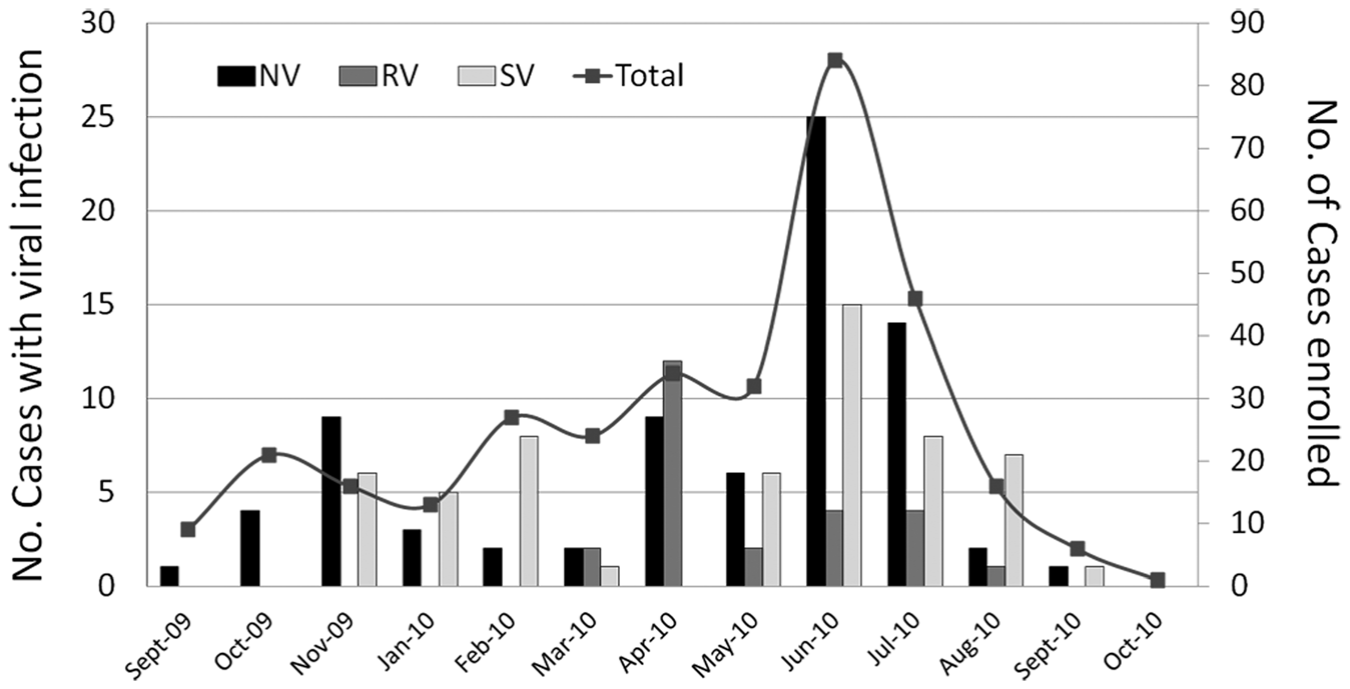
Temporal distribution of norovirus (NV), sapovirus (SV) and rotavirus (RV) infections in children with diarrhea ≤ 5 years of age, after national RotaTeq vaccination in Nicaragua, 2009–2010. Left Y axis; the black, grey and white bars represent monthly frequencies of NV, RV and SV, respectively. Right Y axis; the line represent monthly frequencies of gastroenteritis cases enrolled. The X axis represents seasonality, with the early rainy season typically starting in May and lasting until July.

NV was observed in 79 (24%) of the 330 children with diarrhea investigated in this study. In the hospital setting, NV was observed in 27% (n  =  47) of the children and in the community setting NV was observed in 21% (n  =  32) (OR  =  1.4, *p*  =  0.2). NV was furthermore observed in 15 (26%) of 57 children with severe dehydration (Table 1). NVGE was not gender-dependent and most children with NVGE were less than 24 months of age with a mean age of 12.2 months (median  =  10.1) (Table 1). The major peak for NVGE was observed in June 2010, corresponding to the early rainy season in Nicaragua (Fig. 1). Similar rates of NVGE (24% vs 21%) were observed in RV vaccinated compared to non-vaccinated children.

### The majority of norovirus were of genotype GII.4

Genogroups analysis showed that a total of 71 (90%) NV-positive belonged to GII followed by GI with 7 (9%) and co-infection with both GI:GII in one case (Table 2). To investigate the relative predominance of NV genotypes in hospital and community settings a subset of 55 NV-positive were genotyped by nt sequencing analysis (Table 2). The globally dominant GII.4 genotype was observed in 82% (45/55) of all genotyped cases. The GII.4 genotype was observed in 30 (94%) of the 32 genotyped cases from the hospital and in 15 (65%) of the 23 genotyped cases from the community (Table 2) (OR  =  8.0, *p*  =  0.012). Other detected genotypes were; GII.6, GII.14, GII.9, GI.3 and GI.2 all found in lesser proportions and mainly in the community (Table 1). The pandemic variant GII.4-2006b also known as Minerva was dominant among GII.4 (35/45) and circulated in 2009 and 2010 with high detection rates during the rainy season of 2010. The other detected GII.4 variant, GII.4-2010 (10/45), also known as New Orleans emerged in 2010. The epidemiological peak of GII.4 Minerva and New Orleans variants corresponded with the peak of NVGE and peak of total GE cases (Fig. 1).

**Table 2:** Relative predominance between genetic variants of norovirus, sapovirus and rotavirus among children with gastroenteritis at hospital and community settings in Nicaragua, 2009–2010.

Genetic Variants	Both settings n (%)	Setting		OR^a^ [95%, CI]; *p-value* a
		Hospital n (%)	Community n (%)	
**Norovirus Genogroups**				
GII	71 (90)	44 (94)	27 (84)	4.1 [0.74–22.5]; *0.12*
GI	7 (9)	2 (4)	5 (16)	0.25 [0.04–1.4]; *0.12*
GI:GII	1 (1)	1 (2)	0 (0)	NA^e^
Total	79 (100)	47 (100)	32 (100)	
**Norovirus Genotypes**				
GII.4^b^	45 (82)	30 (94)	15 (65)	8.0 [1.5–42]; *0.012*
GII Non-GII.4^c^	7 (13)	1 (3)	6 (26)	0.09 [0.01–0.82]; *0.017*
GI.3	2 (4)	1 (3)	1(4)	0.71 [0.04–12]; *1.0*
GI.2	1 (2)	0	1 (4)	NA
Total	55 (100)	32 (100)	23 (100)	
**Sapovirus Genogroups**				
GI	26 (46)	14 (52)	12 (40)	2.3[0.67–8.1]; *0.23*
GII	16 (28)	5 (19)	11 (37)	0.39 [0.11–1.4]; *0.21*
GIV	2 (3)	1 (4)	1 (3)	1.2 [0.07–21]; *1.0*
Mixed^d^	5 (9)	3 (11)	2 (7)	NA
Not genogrouped	8 (14)	4 (15)	4 (13)	NA
Total	57 (100)	27 (100)	30 (100)	
**Sapovirus Genotypes**				
GI/1	6 (27)	5 (36)	1 (12)	4.4 [0.42–47]; *0.34*
GI/2	8 (36)	7 (50)	1 (12)	8.0 [0.78–82]; *0.086*
GII/2	4 (14)	1 (7)	3 (25)	0.15 [0.01–1.8]; *0.26*
GII/3	5 (23)	1 (7)	4 (50)	0.1 [0.01–1.08]; *0.056*
Total	23 (100)	14 (100)	9 (100)	
**Rotavirus Genotypes**				
G1P[8]	16 (64)	16 (67)	0 (0)	NA
G3 P[8]	5 (20)	5 (21)	0 (0)	
G4 P[4]	1 (4)	1 (4)	0 (0)	
Not genotyped	3 (12)	2 (8)	1 (100)	
Total	25 (100)	24 (100)	1 (100)	

a Fisher's exact test with unadjusted odds ratio. Relative frequencies of genetic variants in hospital vs community setting. Mixed and non-typed infections were excluded from analysis.

b Only GII.4-2006 Minerva (n  =  35) and GII.4-2009 New Orleans (n  =  10) genetics variants were detected among GII.4.

c GII.6 (n  =  3), GII.14 (n  =  3) and GII.9 (n  =  1).

d GI:GII (n = 3), GI:GIV (n = 1) and GI:GV(n = 1).

e Not applicable.

### Sapovirus was the second major cause of pediatric gastroenteritis

SV was observed in 57 (17%) of the 330 children with diarrhea investigated in this study (Table 1). In the hospital setting, SV was observed in 15% (n  =  27) of the children, and in the community setting SV was observed in 19% (n  =  30) (OR  =  0.76, *p*  =  0.38). Most of the SV were observed in children with no or mild dehydration (48/57; 84%). Similar to NV, SVGE was not gender-dependent, with the majority of children with SVGE (45/57, 79%) being 7–24 months of age (Table 1) (OR  =  2.3, *p*  =  0.02), with the mean age of 12.7 months (median  =  11.8). As for NVGE; the epidemiological peak of SVGE was in June 2010 corresponding to the early rainy season (Fig. 1). SV infections occurred in (51/270) children having received at least one RotaTeq dose and nil (0/14) in non-immunized children (OR  =  6.8, *p*  =  0.08) (Table 1).

The most common SV genogroup was GI (46%), followed by GII (28%), GIV (3%) and mixed infections (9%). In 8 (14%) SV-positive samples, the genogroup could not be determined with the reagents used. To investigate if a particular genetic variant of SV contributed to the unusual high detection rate of SV in the current study, a subset of 23 SV-positives were genotyped by nt sequencing analysis (Table 2). In the hospital setting, GI/1 and GI/2 were the dominant genotypes (12/14), while SV of genotypes GII/2 and GII/3 were mainly found in community cases (7/9) (Table 2).

### Rotavirus gastroenteritis was uncommon but severe in RotaTeq vaccinated children

RV was observed in 25 (8%) of the 330 children with diarrhea investigated. Interestingly, in the community setting, RV was observed in <1% (n  =  1) of the cases whereas in the hospital setting RV was observed in 14% of the cases (n  =  24) (OR  =  24.5, *p* < 0.001), including 16 children with documented RV vaccination. Furthermore, the majority of RV infections were observed in children with severe dehydration (Table 1) (OR  =  11.5, *p* < 0.001). In contrast to NV and SV infections however, there was an equal distribution of RV infections in all age groups (Table 1). Of note is also that the mean age for children with RVGE was high (14.6 months: median  =  10.6). RVGE was observed in 16 (6%) of 270 children vaccinated with RotaTeq and in 2 (14%) of the 14 non-vaccinated children (OR  =  0.38; *p*  =  0.22). RV was also observed in 7 (15%) of 46 children with unknown vaccination status. In RV-positive children having received 3 and 2 RotaTeq doses, the RVGE episode occurred 193 and 65 days (median) post vaccination, respectively.

### Rotavirus of genotypes G1P[8] and G3P[8] infected RotaTeq vaccinated children

Most RV genotypes (16/17), from hospitalized children of Jinotega were G1P[8], whereas the RV genotypes from León were G3P[8] (n  =  4), G9P[4] (n  =  1) and non-typeable (n  =  3) with the reagents used. Full genome sequencing analysis of a G3P[8] RV strain (125L) from León, showed a Wa-like genome (G3-P[8]-I1-R1-C1-M1-A1-N1-T1-E1-H1). Similar genome constellations were previously observed after full genome sequencing of 12 RV-positive from Jinotega, of which 11 were G1P[8] and 1 was G3P[8]; as published elsewhere [26].

### Co-infections with NV and SV

In total 14 (4%) children with GE shed more than one of the virus investigated. Of these, 11 shed SV-NV, 2 SV-RV, and 1 NV-RV, no triple virus infection was observed, but SVGI-SVGII-NVGI was observed. More than 50% (8/14) of the children with co-infections were observed in hospitalized children. Co-infections were confirmed by either secondary PCR or sequencing analysis.

## Discussion

The burden of diarrhea after universal RV vaccination in Nicaragua remains high, despite reduction of RV infections and transmission in the community [12], [16], [18], [33]. In the current study we investigated the role of NV, SV and RV in pediatric gastroenteritis in hospital and community settings in two cities with high RV vaccine coverage (>90%) in Nicaragua.

Our findings show that after RV vaccine implementation, NV has become the leading viral cause (24%) of medically attended acute GE among Nicaraguan children younger than 5 years of age. This is in agreement with recent studies carried out in Finland and USA [34]–[36]. SV was the second most common cause of acute GE (17%), followed by RV (8%). Altogether, these observations indicate that the etiology of viral gastroenteritis at hospital and community settings in Nicaragua has been altered by RV vaccine implementation and that NV and SV strongly contribute to the burden of diarrhea that still persists in the country [17], [18]. Since a viral cause of GE was found in only approximately one half of the children investigated in this study, further surveillance studies of other etiologies are recommended to close this diagnostic gap in these settings [37], [38].

NVGE has been generally described as a mild disease of short duration but new evidence suggests that the illness can be severe, especially among vulnerable populations [39], [40]. The current study is in agreement with such new evidence as 27% of children in the hospital setting had NVGE and many of these required medical attention for mild and severe dehydration. The GII.4 genotypes comprised 94% of all genotyped NV in children in the hospital setting; compared to 65% in the community setting (*p* = 0.012). In a study carried out in Nicaragua in 2005, we observed that most NV cases requiring intravenous treatment were associated with GII.4 (Hunter), and that the same variant was less common in asymptomatic children [22], [41]. Altogether, these observations suggest that besides vulnerable populations, not yet described virulence factors of GII.4 variants contribute to illness severity, a suggestion supported by a recent review of outbreaks associated with GII.4 viruses [39]. A potential bias of our comparison is that community samples were only collected from one city, León, and not Jinotega. However, due to the close geographical location of the cities which have good logistic connections and the long-term nature of the study, it is likely that GII.4 variants circulated in similar proportion in the communities of the two cities.

We further investigated the non-specific effect of RV vaccine against GE from other etiologies, previously suggested in one of the largest clinical trial of RotaTeq [13]. We found no significant differences between rates of NV infection and RV vaccination (24% vs 21%), and NV and SV together, were found in almost a half (24% and 19%, respectively) of the children with diarrhea having received one vaccination dose, with SV only observed in children vaccinated with RotaTeq. These observations strongly suggest that RV-vaccination do not reduce the incidence of NVGE and SVGE.

In previous surveillance studies of pediatric diarrhea in developed and developing countries, prevalence rates of SVGE barely reached 10% [4]–[8]. Despite significant progress on methods for SV detection, it remains poorly recognized as a cause of GE, probably because infection is generally believed mild and few cases need medical care [42]. In the current study, we report that SV infections were the second most common cause (17%) of pediatric GE in the vaccinated population investigated and that SV infections were detected less frequent in children with dehydration as compared to NV and RV. To demonstrate the clinical significance of SaV infections in RV5 vaccinated children; more studies (case – control) should be performed including more non-vaccinated children as well as asymptomatic children. A previous report from Nicaragua found asymptomatic shedding of SaV in ∼9% of children < 5 years of age [8]. A possible explanation for the increased SV prevalence in this study (17%), as compared with a prevalence rate of 12% in 2005–2006 before RV-vaccination in Nicaragua [8] is that there is a higher relative likelihood of finding non-RV enteropathogens in this study where most children had been vaccinated for RV, compared to the time period before RV vaccination. Another reason could be the use of more sensitive methods enhancing detection of most genetic variants. The real-time PCR used in this study was proven to be 10-fold more sensitive than the conventional RT-PCR that uses first generation SV primers (JV33 and SR80) [31], [43].

In line with previous studies we observed a decline of RVGE after introduction of universal RV vaccination [34]–[36], with RV observed in 14% of hospitalized children and less than 1% in community children with diarrhea. This data is also in line with the reduction of incidence of watery diarrhea in the community after RV vaccine introduction in Nicaragua [18]. These observations suggest that RotaTeq implementation has dramatically decreased the number of RVGE in primary care setting in Nicaragua, which is consistent with the low prevalence of RV at environmental level [33].

We investigated whether RV vaccinated children with severe RVGE in the current study were infected with RV strains different from those included in the vaccine. However, genomic analysis of a subset of the strains showed that all RV strains analyzed had genotype specificities typical for human G1P[8] and G3P[8] RVs, similar to genotypes present in the RotaTeq vaccine. This suggests that viral factors alone were not responsible for the vaccine failures in this study population. Another possible explanation could be a short duration of RotaTeq vaccine protection in some children. In this study we observed that RVGE occurred about 6 or 2 months post 3^rd^ and 2^nd^ vaccine dose respectively. A third possible explanation might be that host genetic factors in some children influence vaccine take. A particular attention has now been put on histo-blood group antigens, and remains to be elucidated [44].

To conclude, this study shows that NV has become the leading viral cause of pediatric viral gastroenteritis at hospital and community settings in Nicaragua after universal implementation of RotaTeq vaccine; and that NV together with SV infections strongly contribute to the high burden of diarrhea remaining after RV vaccination.
